# Controlled release of dextrin-conjugated growth factors to support growth and differentiation of neural stem cells

**DOI:** 10.1016/j.scr.2018.10.008

**Published:** 2018-12

**Authors:** Elaine L. Ferguson, Sameza Naseer, Lydia C. Powell, Joseph Hardwicke, Fraser I. Young, Bangfu Zhu, Qian Liu, Bing Song, David W. Thomas

**Affiliations:** a Oral and Biomedical Sciences, School of Dentistry, College of Biomedical and Life Sciences, Cardiff University, Heath Park, Cardiff CF14 4XY, UK; b Cardiff Institute of Tissue Engineering and Repair, Cardiff University, 10 Museum Place, Cardiff, South Glamorgan, CF10 3BG, UK

**Keywords:** BCA, bicinchoninic acid, bFGF, basic fibroblast growth factor (FGF-2), CLSM, confocal laser scanning microscopy, DMAP, 4-dimethylaminopyridine, DMF, *N*,*N*-dimethyl formamide, DMSO, dimethyl sulfoxide, DNA, deoxyribonucleic acid, EDC, 1-ethyl-3-(3-dimethylaminopropyl carbodiimide hydrochloride), EGF, epidermal growth factor, EMEM, eagle's minimum essential media, FCS, fetal calf serum, FPLC, fast protein liquid chromatography, GF, growth factor, GFAP, glial fibrillary acidic protein, GPC, gel permeation chromatography, HAMC, hyaluronan and methyl cellulose, Hep2, human epidermoid carcinoma, MAP2, microtubule-associated protein 2, mNSC, mouse neural stem cell, MTT, 3-(4,5-dimethylthiazol-2-yl)-2,5-diphenyltetrazolium bromide, NSC, neural stem cell, Olig2, oligodendrocyte transcription factor, PBS, phosphate buffered saline, PLGA, poly(lactic-*co*-glycolic acid), SCI, spinal cord injury, sulfo-NHS, N-hydroxysulfosuccinimide, TUNEL, terminal deoxynucleotidyl transferase (TdT) dUTP nick-end labelling, Polymer therapeutics, Biodegradable polymers, Growth factors, Neural stem cells, Controlled release

## Abstract

An essential aspect of stem cell *in vitro* culture and *in vivo* therapy is achieving sustained levels of growth factors to support stem cell survival and expansion, while maintaining their multipotency and differentiation potential. This study investigated the ability of dextrin (~74,000 g/mol; 27.8 mol% succinoylation) conjugated to epidermal growth factor (EGF) and basic fibroblast growth factor (bFGF; or FGF-2) (3.9 and 6.7% w/w protein loading, respectively) to support the expansion and differentiation of stem cells *in vitro via* sustained, controllable growth factor release. Supplementation of mouse neural stem cells (mNSCs) with dextrin-growth factor conjugates led to greater and prolonged proliferation compared to unbound EGF/bFGF controls, with no detectable apoptosis after 7 days of treatment. Immunocytochemical detection of neural precursor (nestin) and differentiation (Olig2, MAP2, GFAP) markers verified that controlled release of dextrin-conjugated growth factors preserves stem cell properties of mNSCs for up to 7 days. These results show the potential of dextrin-growth factor conjugates for localized delivery of bioactive therapeutic agents to support stem cell expansion and differentiation, and as an adjunct to direct neuronal repair.

## Highlights

• Biodegradable dextrin was conjugated to EGF and bFGF to improve stability.
• Sustained release of growth factors potentiated neural stem cell proliferation *in vitro*.
• Dextrin conjugates maintained stem cells in an undifferentiated state.

## Graphical abstract

**Figure:**
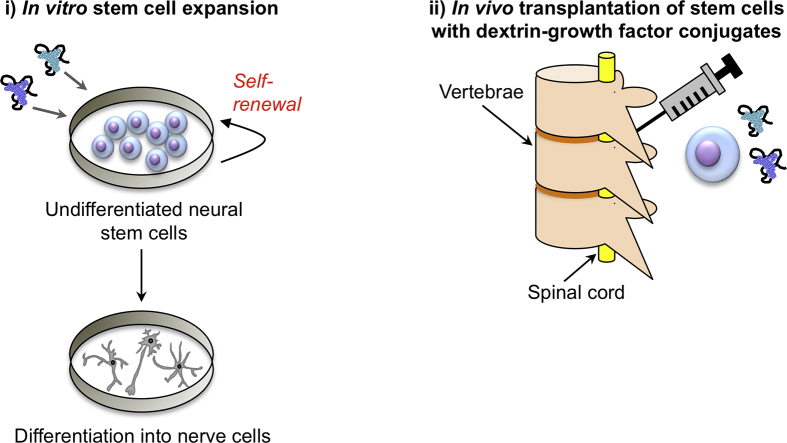

## 1 Introduction

The transplantation of a variety of stem cells (embryonic, neural stem cells (NSC) and non-neural (*e.g.* hematopoietic) stem cells) has been widely investigated as a means of promoting repair and regeneration following injury, due to their ability to self-renew and differentiate into a wide range of cell types. Stem cell therapy shows particular promise as a therapeutic tool to repair and regenerate the nervous system in Parkinson's disease (Kim et al., 2002), neurodegenerative diseases such as Alzheimer's disease (Ager et al., 2015) and following spinal cord injury (SCI) (Liu et al., 2013). However, their clinical application is limited by the need to expand stem cell numbers *in vitro*, while maintaining their multipotency and differentiation potential. The importance of growth factors in maintaining stem cells in an undifferentiated state prior to implantation has necessitated the addition of exogenous growth factors, such as basic fibroblast growth factor (bFGF; also known as FGF-2) and epidermal growth factor (EGF), to *in vitro* culture media to promote cell proliferation, survival and migration and reduce de-differentiation (Ciccolini et al., 2005; Dayer et al., 2007; Sun et al., 2009; Tamama et al., 2010; Meng et al., 2008). However, since exogenous growth factors are rapidly degraded in culture medium (Lotz et al., 2013), standard methods require daily replacement of the medium, making stem cell culture expensive and labor-intensive. Achievement of sustained levels of growth factors has been shown to increase stem cell markers, decrease differentiation markers and increase numbers of stem cells (Lotz et al., 2013). *In vivo*, utilization of stem cell therapies is once again limited by the fundamental role of growth factors in neuronal survival, neurite outgrowth, synaptic plasticity and neurotransmission (Gazdic et al., 2018). Despite 25 years of research and theoretical potential, recombinant growth factors have had limited success in clinical practice; principally due to their rapid clearance from the target site by diffusion and lymphatic drainage, and premature inactivation by proteolytic enzymes and reactive oxygen species *in vivo*. As a result, whilst clinical trials have demonstrated the technique to be safe, attempts to induce healing using stem cell implantation have shown only limited success clinically, with typical survival rates of transplanted stem cells between 0.5 and 38% (Walsh and Midha, 2009; Piltti et al., 2015).

Polymer therapeutics are increasingly being developed as innovative therapies for tissue repair and regeneration (Duncan and Vicent, 2013). We have previously developed and characterized dextrin-growth factor conjugates using EGF as a model growth factor to promote tissue repair (Hardwicke et al., 2008; Hardwicke et al., 2010a; Hardwicke et al., 2010b; Hardwicke et al., 2011). Such conjugates exhibit sustained and controlled degradation of the dextrin component in the presence of physiological concentrations of α-amylase, an enzyme that is widely distributed in extracellular fluids and serum (30–110 IU/L), leading to sustained release of free EGF, protein unmasking and restoration of bioactivity to the level seen for unmodified EGF (Hardwicke et al., 2008). In this wound healing model, we demonstrated not only increased dermal cell proliferation and migration with the dextrin-EGF, but also that the duration of effect was longer than that observed with free EGF (Hardwicke et al., 2008; Hardwicke et al., 2010a). In an *ex vivo* organotypic model of re-epithelialisation, dextrin-conjugated EGF was as effective as free EGF at 10× lower doses (Hardwicke et al., 2010b), and in an *in vivo* model of impaired wound healing in diabetic (db/db) mice, topical application of dextrin-EGF significantly enhanced dermal wound healing (Hardwicke et al., 2011). The potential for using such polymer therapeutics to support stem cell therapy is clear, to both improve the efficiency and cost-effectiveness of *in vitro* stem cell expansion, and increase the survival, integration and differentiation of transplanted stem cells *in vivo*. Encapsulation of growth factors, such as FGF-2 (sold commercially as a growth factor supplement (StemBeads®)) and vascular endothelial growth factor (VEGF), into microparticles, has been tested as a means of achieving sustained release for improved *in vitro* stem cell culture (Lotz et al., 2013) and *in vivo* survival (Aday et al., 2017). The lack of a mechanism to control the release/delivery of the growth factors in these systems may, however, limit their use clinically.

The aim of this study was, therefore, to investigate the ability of two model dextrin-growth factor conjugates (dextrin-EGF and dextrin-bFGF) to support *in vitro* stem cell proliferation and differentiation by sustained, controllable growth factor release and demonstrate their potential as a supplement for stem cell therapy for pathologies such as SCI. Dextrin-EGF and -bFGF conjugates have been synthesized and characterized using fast protein liquid chromatography (FPLC), gel permeation chromatography (GPC) and a bicinchoninic acid (BCA) assay. Their ability to promote proliferation, prevent apoptosis and regulate the differentiation of mouse neural stem cells (mNSCs) was investigated in these studies.

## 2 Materials and methods

### 2.1 Materials and cells

Type 1 dextrin from corn (Mw ~ 51,000 g/mol) was from ML laboratories (Keele, UK). Recombinant human EGF and recombinant human bFGF were from Prospec-Tany Technogene Ltd. (Rehovot, Israel). Anhydrous *N*,*N*-dimethyl formamide (DMF), 1-ethyl-3-(3-dimethylaminopropyl carbodiimide hydrochloride) (EDC), copper (II) sulfate pentahydrate 4% *w*/*v* solution, 3-(4,5-dimethylthiazol-2-yl)-2,5-diphenyltetrazolium bromide (MTT), amylase from human saliva, and bicinchoninic acid (BCA) solution were all from Sigma-Aldrich (Poole, UK). Sodium acid phosphate, sodium phosphate, sodium chloride, N-hydroxysulfosuccinimide (sulfo-NHS) and 4-dimethylaminopyridine (DMAP) were from Fisher Scientific (Loughborough, UK). Pullulan gel filtration standards (Mw = 11,800–788,000 g/mol) were from Polymer Laboratories (U.K.). Unless otherwise stated, all chemicals were of analytical grade. All solvents were of general reagent grade (unless stated) and were from Fisher Scientific (Loughborough, UK).

Human epidermoid carcinoma (Hep2; ATCC no.: CCL-23) cells and Eagle's minimum essential media (EMEM) with l-glutamine and Earle's balanced salt solution adjusted to contain 1.5 g/L sodium bicarbonate, non-essential amino acids and sodium pyruvate were purchased from LGC Promochem (Teddington, UK). Primary neural stem cells (NSCs) were derived from the embryonic mouse cortex on day E14 and cultured using the neurosphere method described previously (Meng et al., 2007). Cells were screened and found to be free of mycoplasma contamination before use. DMEM/F12 media with l-glutamine, N2 supplement, fetal calf serum (FCS), 0.05% w/w trypsin-0.53 mM EDTA, antibiotic-antimycotic solution (containing penicillin G (10,000 units/mL), streptomycin (10,000 μg/mL) and amphotericin B (25 μg/mL)) and StemPro®Accutase® were obtained from Invitrogen Life Technologies (Paisley, UK). EGF and bFGF for routine cell maintenance were from Peprotech (London, UK). Laminin and optical grade dimethyl sulfoxide (DMSO) were from Sigma-Aldrich (Poole, UK). Poly-d-lysine was from Millipore (Consett, UK).

### 2.2 Synthesis of dextrin-growth factor conjugates

Succinoylated dextrin (27.8 mol% succinoylation, 74,000 g/mol) was synthesized according to the method of Hreczuk-Hirst et al. (Hreczuk-Hirst et al., 2001) and characterized as described by Ferguson and Duncan (2009). The succinoylated dextrin intermediate was conjugated to EGF using EDC and sulfo-NHS as coupling agents, as described by Hardwicke et al. (Hardwicke et al., 2010b). For dextrin-bFGF conjugation, a modified version of the same method was employed, however, since bFGF's molecular weight is 2.8 times greater than EGF, a molar ratio of 1 bFGF to 1 dextrin was used. Briefly, succinoylated dextrin (11.8 mg, 2.3 × 10^−7^ mol) was dissolved under stirring in ddH_2_O (800 μL) in a 10 mL round-bottomed flask. To this, EDC (2 mg, 1.0 × 10^−5^ mol) and sulfo-NHS (2 mg, 9.2 × 10^−6^ mol) were added and the solution was stirred for 15 min. Next, bFGF (4 mg, 2.3 × 10^−7^ mol) was added and the pH adjusted to 8.0 by dropwise addition of NaOH (0.5 M) before allowing the reaction to proceed for 2 h. The products were purified using FPLC, prior to lyophilization and storage at −20 °C.

Conjugates were characterized in respect of free protein by FPLC (ÄKTA Purifier; GE Healthcare, UK) using a pre-packed Superdex 75 10/300 GL column with a UV detector and data analysis using Unicorn 5.20 software (GE Healthcare, UK). Samples (200 μL) were injected into a 100 μL loop with a 0.1 M phosphate buffer with 0.15 M sodium chloride (PBS), pH 7.4 at 0.5 mL/min. The molecular weight was estimated relative to protein standards. The total protein content of the conjugate was determined by the BCA assay.

### 2.3 Degradation of dextrin-EGF conjugates by amylase

To compare the concentration-dependent rate of amylase degradation of dextrin-EGF conjugates, solutions (1 mg/mL in PBS, pH 7.4) were prepared containing amylase (0, 20, 93 IU/L in PBS) and incubated at 37 °C for up to 168 h. At various time-points, samples (150 μL) were taken, immediately snap frozen in liquid nitrogen to stop the reaction and then stored at −20 °C until analysis. The samples were thawed and analyzed by FPLC to determine the change in % free EGF over time by comparing the ratio of the area under the curve corresponding to free and dextrin-conjugated EGF.

### 2.4 Cell culture

All cells were routinely grown in a 75 cm^3^ tissue culture flasks and incubated at 37 °C/5% CO_2_. HEp2 cells were cultured in EMEM media containing heat-inactivated FCS (10% v/v) and antibiotic-antimycotic solution. HEp2 cell monolayers were detached from the surface using trypsin-EDTA solution. mNSCs were cultured in DMEM/F12 media containing N2 (1% v/v), EGF (10 ng/mL) and bFGF (10 ng/mL). Neurospheres were digested to single cells using StemPro® Accutase®.

### 2.5 Evaluation of cell proliferation by MTT assay: Restoration of biological activity by amylase

An MTT assay (Mosmann, 1983) was used to assess cell proliferation (72 h incubation) in HEp2 cells in the presence of dextrin-EGF conjugate that had been pre-exposed to varying physiological concentrations of amylase. Here, dextrin-EGF conjugate (50 ng/mL EGF equiv.) was incubated with amylase (0, 20, 93 IU/L) in serum-free media for 24 h at 37 °C, prior to adding to the cells. Meanwhile, cells were seeded into the inner wells of sterile 96-well microtiter plates (2.5 × 10^4^ cells/mL) in 0.1 mL/well of serum-free media to excluded interference from other sources of EGF. Outer wells were filled with sterile PBS (0.1 mL/well) and cells were allowed to adhere for 24 h. The medium was then removed and test compounds (0.2 μm filter-sterilized) were added. After 67 h, MTT (20 μL of a 5 mg/mL solution in PBS) was added to each well and the cells were incubated for a further 5 h. The medium was then removed and the precipitated formazan crystals solubilized by addition of DMSO (100 μL). Absorbance was measured at 550 nm after 30 min using a microtiter plate reader. Cell viability was expressed as a percentage of the viability of untreated control cells.

### 2.6 Monolayer response model

Cell proliferation was assessed daily over 7 days using an MTT assay as previously described (Mosmann, 1983). Briefly, cells were seeded into the inner wells of sterile poly-d-lysine/laminin coated 96-well microtiter plates (5 × 10^4^ cells/mL) in 0.1 mL/well of media containing N2 (1% v/v), EGF (10 ng/mL) and bFGF (10 ng/mL) at 37 °C/5% CO_2_. Outer wells were filled with sterile PBS (0.1 mL/well). Cells were allowed to adhere for 24 h. The medium was then removed and filter-sterilized (0.2 μm) test compounds dissolved in DMEM/F12 media containing N2 supplement were added to the cells as follows: (1) control (no growth factors); (2) EGF (10 ng/mL) and bFGF (10 ng/mL); (3) dextrin-EGF (10 ng/mL EGF equiv.) and bFGF (10 ng/mL); (4) EGF (10 ng/mL) and dextrin-bFGF (10 ng/mL bFGF equiv.); and (5) dextrin-EGF (10 ng/mL EGF equiv.) and dextrin-bFGF (10 ng/mL bFGF equiv.). Proliferation was monitored daily using an MTT assay, as described previously, and cell viability was expressed as a percentage of the absorbance reading on day 0. Data were normalized to the presence or absence of EGF + bFGF (EGF + bFGF and EGF/bFGF-free medium were set at 100% and 0%, respectively). Pictures of the wells of a 24-well plate treated as described above were taken immediately after addition of treatments (*t* = 0) and daily thereafter using a Canon PC1234 digital camera, (Japan) attached to a Nikon Eclipse TS100 light microscope (UK).

### 2.7 Three-dimensional sphere response model

To study cell proliferation effects in a 3-dimensional model, cells were seeded into the inner wells non-coated 96-well microtiter plates (5 × 10^4^ cells/mL) in 0.1 mL/well of media containing N2 (1% v/v), EGF (10 ng/mL) and bFGF (10 ng/mL) at 37 °C/5% CO_2_. Outer wells were filled with sterile PBS (0.1 mL/well). Treatments were added after 24 h as previously described and proliferation was monitored daily using an MTT assay and visually by a microscope. Data were normalized to the presence or absence of EGF + bFGF (EGF + bFGF and EGF/bFGF-free medium were set at 100% and 0%, respectively). *In vitro* cell proliferation was also studied by analysis of neurosphere number and mean diameter. Pictures of the wells of a 96-well plate treated as described above were taken immediately after addition of treatments (t = 0) and daily thereafter using a Canon PC1234 digital camera, (Japan) attached to a Nikon Eclipse TS100 light microscope (UK). The number of neurospheres and their average diameter was screened using random sampling (1 visual field per well, n = 6) and quantified using ImageJ software (Rasband, 1997–2015). Neurospheres were classed based on their mean diameter: small (50–100 μm), medium (100–200 μm) and large (≥200 μm). Neurospheres on the edges of each image were excluded from analysis, so that only completely visualized spheres were quantified.

### 2.8 Evaluation of apoptosis in mNSC

To assess apotosis of the mNSC, cells were seeded into the inner wells of 24-well microtiter plates (5 × 10^4^ cells/mL) in 0.6 mL/well of media containing N2 (1% v/v), EGF (10 ng/mL) and bFGF (10 ng/mL) at 37 °C/5% CO_2_. Outer wells were filled with sterile PBS (2 mL/well). Treatments (1 mL) were added after 24 h as previously described and apoptosis was analyzed using a DeadEnd™ Fluorometric TUNEL system (Promega, UK) following the manufacturer's instructions. Cells were counterstained with DAPI contained in Vectashield mounting medium (Vector Laboratories, Peterborough, UK) and imaged using an ultra violet (UV) microscope (Olympus AX70 with a Digital Eclipse DX M1200 digital camera attachment (Japan). The images were captured using the Automatic Camera Tamer (ACT-1) control software (Nikon Digital, Japan) at λ_ex_ = 373 nm, λ_em_ = 456 nm (DAPI) and λ_ex_ = 490 nm, λ_em_ = 520 nm (FITC). The number of TUNEL-positive cells (defined as a TUNEL signal that overlapped with the DAPI stained nuclei) was screened using random sampling (2 visual fields per well, n = 2) and expressed as the percentage of TUNEL-positive cells out of the total number of mNSCs counted.

### 2.9 Immunofluorescent analysis of mNSC properties

Following the same plate set-up procedure as the apoptosis assay, cells were prepared for immunofluorescent analysis immediately after addition of treatments (*t* = 0) and on days 5 and 7. Briefly, mNSCs were fixed with freshly prepared paraformaldehyde solution (4% *v*/*w* in PBS) for 20 min, permeabilized with 0.2% Triton X-100 for 10 min and incubated in blocking solution (1% BSA in PBS) for 1 h at room temperature, followed by incubation with primary antibodies at 4 °C overnight. After extensive washing with the blocking solution, cells were incubated with secondary antibodies at 37 °C for 1 h. Then, mNSCs were mounted in Vectashield mounting medium with DAPI. All antibodies were diluted in blocking solution. The primary antibodies used were nestin, a monoclonal marker for neural stem cells (1:100, Santa Cruz SC-33677); MAP2, a polyclonal antibody specific for neurons (1:250, Millipore AB5622); GFAP, a monoclonal antibody specific for astrocytes (1:500, Millipore 04–1062) and Olig2, a polyclonal antibody specific for oligodendrocytes (1:500, Millipore AB9610). The secondary antibodies were Alexa 594-conjugated goat anti-mouse and anti-rabbit IgGs, (1:500; Life Technologies, UK).

Confocal fluorescence microscopy was performed using an inverted Leica SP5 Confocal laser scanning microscopy (CLSM) and a x63 oil immersion lens, with a scan speed of 400 Hz. Data was collected using Leica LAS AF software and exported as tagged image files (TIF); at least 3 representative images were obtained for each sample. Typical results are shown. Merged images were generated using the Leica LAS AF software. To determine the percentage of the nestin/GFAP/MAP2/Olig2-positive cells, the numbers of DAPI-labeled cell nuclei and those double-labeled with AlexaFluor 594 were counted. Nestin/GFAP/MAP2/Olig2 expression was qualified and quantified using random sampling.

### 2.10 Statistical analysis

Data are expressed as mean ± the error, calculated as either standard deviation (SD) where n = 3, or standard error of the mean (SEM) where n > 3. Statistical significance was set at *p* < .05 (indicated by *). Evaluation of significance was achieved using a one- or two-way analysis of variance (ANOVA) followed by Bonferroni *post hoc* tests that correct for multiple comparisons. All statistical calculations were performed using GraphPad Prism, version 6.0 h for Macintosh, 2015.

## 3 Results

Dextrin-EGF and -bFGF conjugates used in these studies had an average protein content of 3.9 (equivalent to 3 dextrin: 1 EGF) and 6.7% w/w protein (equivalent to 4.7 dextrin: 1 bFGF), respectively. The average molecular weight was 190,000 and 180,000 g/mol, for dextrin-EGF and –bFGF, respectively. FPLC indicated that the free protein content of the conjugates was always <1%. Neither dextrin-EGF or dextrin-bFGF conjugates were cytotoxic towards mNSC, up to 500 ng/mL growth factor equiv. (measured by MTT assay, 72 h incubation; *data not shown*).

### 3.1 Effect of amylase concentration on dextrin-EGF conjugate stability and activity

**Fig. 1:**
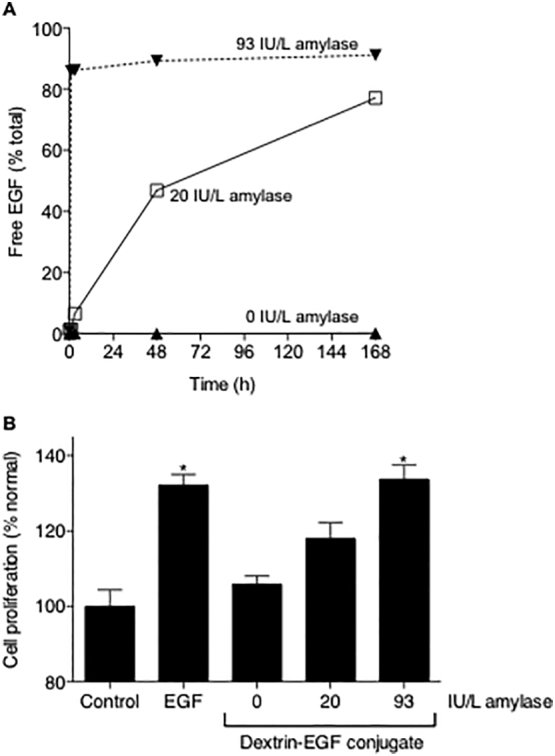
Stability of dextrin-EGF conjugate in physiologically relevant concentrations of amylase. (A) Release of EGF from dextrin-EGF conjugates (1 mg/mL) in the absence and presence of amylase (20–93 IU/L in PBS) at 37 °C (measured by FPLC). Data is expressed as the percentage of total EGF (n = 1). (B) Proliferation of HEp2 cells (72 h incubation) in the presence of EGF or the dextrin-EGF conjugate (6.25 ng/mL EGF equiv.) with amylase (0–93 IU/L). Data represents mean ± SD, n = 6. * indicates significance *p* < .01 compared to control (no GF).

FPLC analysis of amylase-treated dextrin–EGF conjugate showed a time- and concentration-dependent increase in concentration of free EGF, indicating release of EGF from the conjugate due to amylase degradation of dextrin. In these experiments, 93 IU/L amylase released the most free EGF (~91.1%) after 168 h incubation (Fig. 1A), however, significant EGF release was observed at amylase concentrations similar to those found in cerebrospinal fluid (20 IU/L) (77.1% after 168 h incubation).

In the HEp2 cell proliferation model, dextrin-EGF conjugate activity (in the absence of amylase) was not significantly different to control (*p* > .05) but was significantly lower than free EGF (*p* < .01) (Fig. 1B). When conjugates were pre-incubated with amylase, HEp2 cell proliferation was restored in a concentration-dependent manner, whereby amylase concentrations of 93 IU/L produced no significant difference in cell proliferation after 72 h compared to free EGF (*p* > .01).

### 3.2 Stimulation of mNSC proliferation

**Fig. 2:**
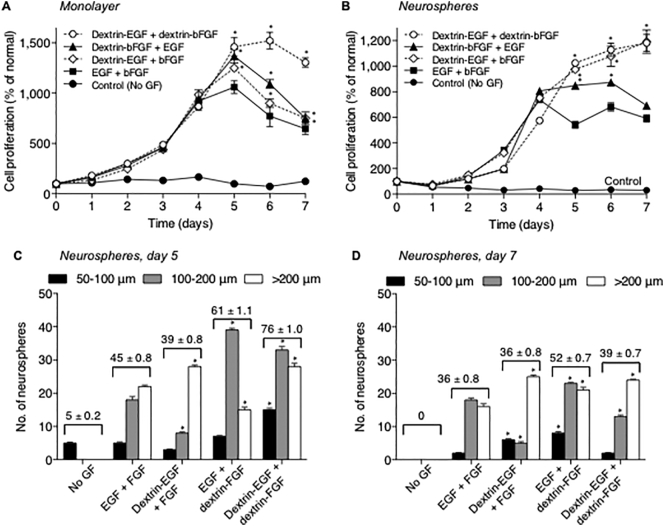
Proliferation of mNSC over 7 days. Growth curves (using the MTT assay) of cells grown as (A) a monolayer and (B) neurospheres, when cells were grown in growth factor-free medium (control), or in the presence of free- or dextrin-conjugated EGF and bFGF. Data represent mean ± SEM, n = 18. Categorization of neurospheres based upon diameter: small (100 μm), medium (100–200 μm) and large (> 200 μm) on (C) day 5 and (D) day 7. Data represent mean ± SEM, n = 6. Where error bars are invisible they are within size of data points. *Indicates significance *p* < .0001 compared to EGF + bFGF. Values above bars correspond to mean total neurosphere number ± SEM, n = 6.

To quantify the mitogenic activity of dextrin-growth factor conjugates, the proliferation profile of mNSCs during incubation with free and/or dextrin-conjugated growth factors was examined for 7 days (Fig. 2). When adherent cells were grown in the absence of growth factors, no proliferation was observed (Fig. 2A). In contrast, proliferation of mNSC exposed to unbound growth factors stimulated cell proliferation which peaked at day 5, with a corresponding decrease in cell density and morphological changes thereafter. Supplementation with one ‘free’ (unconjugated) and one conjugated growth factor showed a similar proliferation profile, but with more viable cells from day 5 than was observed with free growth factors. The addition of dextrin-EGF and -bFGF conjugates led to the greatest number of viable cells and prolonged proliferation, which only showed a drop in cell viability after day 6, when confluency had been reached (*p* < .0001 compared to EGF + bFGF).

Similarly, when cells were grown in uncoated plates, to allow neurosphere formation, supplementation with dextrin-EGF and -bFGF conjugates led to the greatest and most prolonged proliferation of mNSCs, that did not reach a peak within 7 days (Fig. 2B). Treatment with unconjugated growth factors led to maximal cell viability on day 4, which was lower than the other treatments, and followed by a gradual decrease in viable cell numbers thereafter. Neurospheres grown in the presence of dextrin-EGF + bFGF showed comparable proliferation to the combined dextrin-growth factors, while the EGF + dextrin-bFGF combination was only modestly better than free growth factors. When neurospheres were categorized according to size, those grown in the presence of dextrin-conjugated growth factors were more abundant than those cultured with unbound growth factors (Fig. 2C,D). Neurospheres grown in the presence of the dextrin-conjugated growth factors also appeared more dense (*data not shown*).

### 3.3 Detection of apoptosis in growth factor-treated mNSC

**Fig. 3:**
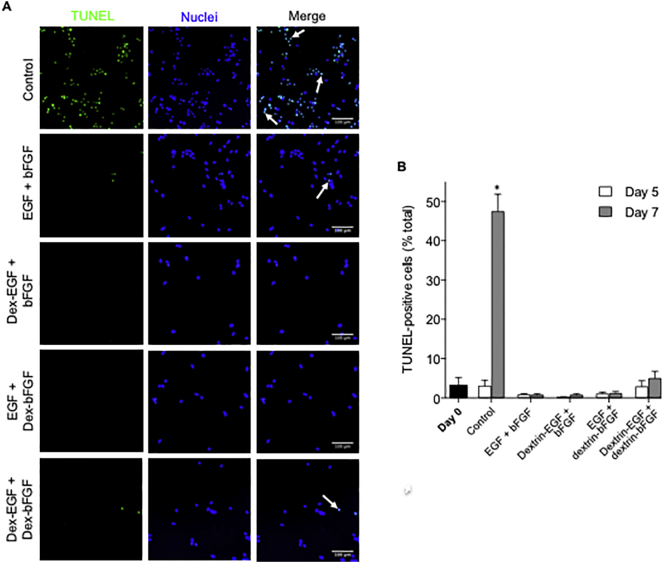
TUNEL assay to detect intranuclear DNA fragmentation in apoptotic mNSC. (A) Visualization on day 7 and (B) quantification on days 0, 5 and 7 of TUNEL-positive cells (indicated with white arrows). In all microscopy images, the left-hand column represents data shown in the green (FITC) channel, the middle column represents data shown in the blue (DAPI) channel and the data shown in the right-hand column represents merged data. Scale bar = 100 μm. Data represent mean ± SEM, n = 8. * indicates significance *p* < .0001 compared to day 0. (For interpretation of the references to colour in this figure legend, the reader is referred to the web version of this article.)

TUNEL labeling was used to study apoptosis of mNSC grown in coated plates at the cellular level. In the absence of growth factors, numbers of TUNEL-positive cells increased significantly from day 0 to 7 (47.4% ± 12.6 (SD) TUNEL-positive cells, *p* < .0001); cells exhibited the characteristic fragmented nuclei (Fig. 3). Supplementation with free or dextrin-conjugated growth factor treatments did not trigger significant cell apoptosis after 7 days (<5% TUNEL-positive cells).

### 3.4 Evaluation of mNSC characteristics by immunofluorescence

**Fig. 4:**
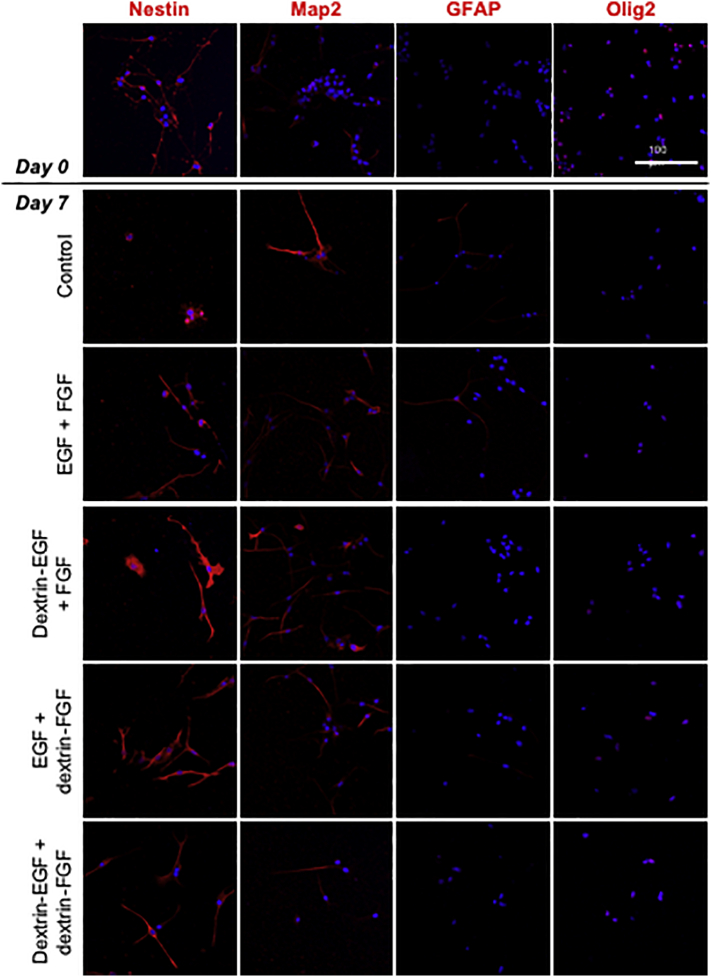

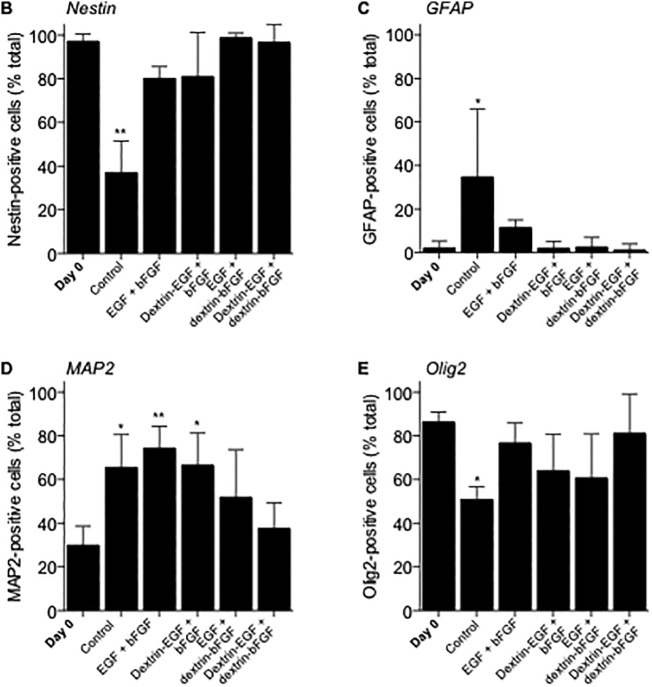
(A) Detection of NSC and differentiation markers. Experiments conducted in mNSC grown in growth factor-free medium (control), or in the presence of free- or dextrin-conjugated EGF and bFGF by visualization of nestin/GFAP/MAP2/Olig2-positive cells on days 0, 5 and 7. In all microscopy images, all figures represent merged data from the red (Alexa Fluor® 594) and blue (DAPI) channels. Scale bar = 100 μm. Quantification of (B) nestin, (C) MAP2, (D) GFAP and (E) Olig2-positive cells on day 7. Data represent mean ± SD, n = 3. *Indicates significance *p* < .01 and **indicates significance *p* < .001, compared to day 0. (For interpretation of the references to colour in this figure legend, the reader is referred to the web version of this article.)

To investigate the stem cell characteristics of mNSCs, nestin expression was visualized in adherent cells by immunocytochemistry (Fig. 4A). Cells grown in the absence of any growth factors (control) showed reduced nestin-positivity after 7 days (37.1% ± 14.5 (SD) nestin-positive cells, *p* < .0001) (Fig. 4B). No statistically significant alteration in the proportion of nestin-positive cells was observed in any of the growth factor-treated groups, however, there was a trend for slightly reduced nestin expression in cells treated with unconjugated bFGF (EGF + bFGF (80.2% ± 5.4 (SD) nestin-positive cells) and dextrin-EGF + bFGF (81.1% ± 20.1 (SD) nestin-positive cells) compared to dextrin-conjugated bFGF (EGF + dextrin-bFGF (98.8% ± 2.1 (SD) nestin-positive cells) and dextrin-EGF + dextrin-bFGF (96.7% ± 8.2 (SD) nestin-positive cells) after 7 days.

To determine whether mNSCs maintained their ability to differentiate in the presence of dextrin-conjugated growth factors, the expression of neuron, astrocyte and oligodendrocyte lineage markers was assessed by confocal fluorescent microscopy of immune-labeled cells (Fig. 4). Control cells, grown in the absence of any growth factors, showed elevated expression of MAP2 (*p* < .001) and GFAP (*p* < .01), and reduced expression of Olig2 (*p* < .001), compared to the cells on day 0 (Fig. 4C,D,E). Cells treated with unconjugated bFGF showed increased differentiation to a neuronal lineage (EGF + bFGF (74.4% ± 10.0 (SD), *p* < .0001) and dextrin-EGF + bFGF (60.6% ± 14.7 (SD), *p* < .001) *vs.* control (30.0% ± 10.0 (SD)) MAP2-positive cells), whereas supplementation with the dextrin-growth factor combination conjugates did not alter marker expression after 7 days incubation. Combining free EGF with dextrin-bFGF conjugates had no effect on expression of any of the differentiation markers.

## 4 Discussion

Stem cell therapy could potentially offer life-altering improvements in function, mobility and pain for patients with SCI, due to their ability to differentiate into the many different cell types that make up the spinal cord. Ideally, a stem cell therapy for treating neuronal injuries should be generated using efficient, cost-effective strategies to produce high numbers of homogeneous undifferentiated stem cells. Clinically, the stem cell therapy must be capable of replacing injured cells, to produce aligned fibers of differentiated cells, with minimal scar tissue formation. Stem cells would be administered in a single (or infrequent) operative procedure in a medium that can support proliferation and differentiation for an extended period by sustained delivery of nutrients and growth factors (Coelho et al., 2012). Here, we have shown that the use of biodegradable dextrin-bFGF and -EGF conjugates can increase and prolong the proliferation of mNSC while simultaneously inhibiting apoptosis. Furthermore, mNSCs retain their stem cell properties and demonstrate the ability to differentiate into nerve cells. These observations demonstrate, not only, that dextrin-growth factor conjugates may be used to support *in vitro* stem cell proliferation and differentiation *via* sustained, controllable growth factor release, but may, in the future, be used to improve the delivery, and ultimate fate, of stem cells in transplantation-based repair of SCI and other neuronal injuries.

Duncan et al. have previously shown that controlled reinstatement of protein activity is achieved by degradation of dextrin by physiological (serum) concentrations of amylase (Duncan et al., 2008). However, since cerebrospinal fluid reportedly contains ~12–17 IU/L amylase (Lahiri, 1973) (compared to 30–110 IU/L in serum), our first experiments investigated whether controlled release and reinstatement of growth factor activity could be achieved at these lower amylase concentrations, using dextrin-EGF as a model (Fig. 1). Here, a concentration-dependent increase in rate and extent of EGF release in the presence of low concentrations of amylase was observed; mirrored by the restoration of HEp2 cell proliferation following pre-incubation with equivalent concentrations of amylase. This slower release of growth factor from the conjugate at lower amylase concentrations may be clinically useful, as stem cells administered to treat SCI would take several weeks to generate a functional nerve fiber. Similarly, exposure of mNSC to growth factors stimulated cell proliferation, which was greater and more prolonged when growth factors were conjugated to dextrin; demonstrating the increased stability and sustained release of growth factors that polymer conjugation confers. Cell culture media in these experiments was not supplemented with amylase, since this is not routinely added to stem cell culture media, which allowed us to demonstrate the effectiveness of dextrin-conjugated growth factors as stem cell media supplements for the expansion of stem cells. Preliminary studies were undertaken with the addition of exogenous amylase, in an attempt to reproduce levels observed in acute and chronic inflammation observed in neuronal injury and degenerative disease. Importantly, it was evident that the release and activity of the dextrin-conjugated growth factors occurs in the absence of amylase (supplementary figs. S1,S2) and that the effects of the conjugates were not significantly reduced. This finding may reflect enzymatic activity of the cells *e.g.* lysosomal glycosidase production. The addition of exogenous amylase led to increased stem cell numbers compared to the amylase-free treatment that decreased or plateaued in 2D and 3D cultured cells, respectively, after day 5. Based on these results, it is envisaged that the use of dextrin-conjugated growth factors could significantly improve the efficiency of not only stem cell maintenance and expansion (reducing the necessity for growth factor supplementation by up to 7-fold), but also could be employed clinically at inflamed sites.

It is well known that EGF and bFGF are essential for the proliferation and expansion of neural stem cells (Gritti et al., 1996; Kornblum et al., 1990; Morrison et al., 1988; Morrison et al., 1987; Kojima and Tator, 2002). Recent studies have shown that sustained growth factor levels decrease spontaneous differentiation and increase proliferation, yet this is not achieved in routine stem cell maintenance, even with daily culture medium replacement (Lotz et al., 2013). For example, Lotz et al. (Lotz et al., 2013) showed that FGF-2 levels fell to below 50% of the initial concentration within 4 h, and <25% remained after 24 h, presumably due to denaturation of FGF-2 by heat (Chen et al., 2012), proteolytic enzymes and growth factor-solvent interactions. Consequently, encapsulation into a polyelectrolyte-modified 2-hydroxyethyl methacrylate hydrogel (Galderisi et al., 2013), poly(lactic-*co*-glycolic acid) (PLGA) microspheres (Lotz et al., 2013) and multi-trilayered nanofilms (Han et al., 2017) have been investigated to achieve controlled release of FGF-2 in stem cell culture media. Controlled release of growth factors, as a means of enhancing the proliferation of cells in the central nervous system, has also principally involved incorporation into polymeric hydrogels (Gupta et al., 2006; Kapur and Shoichet, 2004; Kang et al., 2009; Jimenez Hamann et al., 2005). Generally, these systems are designed to release all entrapped growth factors at the same rate (either in an initial burst or within a few days), however, the physiological growth factor signalling cascade is much more complex. Consequently, Bauman et al. developed a composite drug delivery system for independent delivery of multiple drugs entrapped within an injectable hyaluronan (2% w/w) and methyl cellulose (7% w/w) (HAMC) hydrogel containing PLGA nano- and microparticles (Baumann et al., 2009). In a model system designed to treat SCI, rapid, diffusion-controlled release of neuroprotective molecules (*e.g.* bFGF) from the HAMC hydrogel was achieved over 1–4 days, while slow release of neuroregenerative molecules (*e.g.* EGF) over 28 days was attained by entrapment within the PLGA particles. This concept of sustained combination therapy mimics the physiological release of growth factors and could usefully applied to the dextrin- growth factor conjugates developed in these studies. Modification of dextrin's molecular weight and degree of succinoylation have been demonstrated to control the polymer's degradation rate (Duncan et al., 2008), thereby altering the release rate of growth factors. Conjugation of colistin to 1 mol% succinoylated 7500 g/mol dextrin led to ∼80% drug release by amylase degradation within 48 h (Ferguson et al., 2014), compared to 52.7% after 168 h for EGF conjugated to 19 mol% succinoylated 42,000 g/mol dextrin (Hardwicke et al., 2010b). Since the desired exposure to bFGF is days (*vs.* weeks for EGF), this could readily be achieved by its conjugation to a lower molecular weight dextrin with less modification.

Growth factor withdrawal is widely recognised as a trigger for programmed cell death, due to mitochondrial cytochrome C release and caspase activation (Letai, 2006). It therefore follows, that if exogenous growth factors are depleted or degraded in the cell environment, reduced stem cell viability would be accompanied by apoptosis. We, therefore, used a TUNEL assay to detect apoptotic cells during incubation with free and dextrin-conjugated growth factor for 7 days. As expected, mNSCs grown in the absence of any growth factors (control) showed significant apoptosis after 7 days, however, while cell viability of free growth factor-treated mNSCs was clearly reduced after 7 days compared to dextrin-conjugated growth factors, apoptosis was not induced in any of the treatment groups. While these results suggest that free growth factors were not depleted or degraded over the 7-day incubation period, it is important to note that the TUNEL assay detects extensive DNA fragmentation which occurs only in the late stages of apoptosis (Kyrylkova et al., 2012). It is, therefore, possible that cells treated with unconjugated growth factors were in the early stages of apoptosis due to growth factor depletion, that would be undetectable in this assay. Moreover, in addition to apoptosis, growth factor deprivation may promote a death autophagy-like mechanism characterized by vacuole development, which is also undetectable by conventional TUNEL assay (Cardenas-Aguayo Mdel et al., 2003).

Indiscriminate differentiation of neural stem cells occurs in response to growth factor withdrawal (Johe et al., 1996), therefore, it was hypothesized that sustained release of growth factors from polymer conjugates could suppress differentiation and maintain a homogeneous population of multipotent stem cells. As expected, in these experiments, mNSCs showed dramatically reduced expression of the neural progenitor-associated marker nestin following growth factor withdrawal, associated with initiation of differentiation. In contrast, expression of nestin was unaltered in all the growth factor-treated mNSC treatment groups, suggesting that the cells had retained their neural stem cell identity over the 7-day incubation period. However, immunocytochemical evaluation demonstrated that, while addition of dextrin-conjugated EGF and bFGF did not change the expression of neuron, astrocyte and oligodendrocyte differentiation markers after 7 days, mNSCs treated with free growth factors showed some altered expression of differentiation markers, suggesting that growth factor depletion had occurred, presumably due to proteolytic degradation. GFAP expression was also significantly elevated in the growth factor-free mNSCs but remained unaltered in all other treatment groups. Although GFAP-expressing astrocytes may enhance repair following SCI, they also have a significant role in the formation of glial scar tissue (Sofroniew and Vinters, 2010), which inhibits functional recovery and creates a barrier to regeneration (Silver and Miller, 2004). Clinically, these alterations may impede regeneration of a functional spinal cord. Interestingly, the presence of dextrin-conjugated bFGF maintained mNSC MAP2 expression, while unconjugated bFGF led to increased MAP2 expression, indicating that this growth factor may benefit from sustained, controlled release by dextrin conjugation. Olig2 plays an important role in oligodendrocyte formation, however, this factor has also been identified as a multifunctional regulator of NSC self-renewal during expansion (Mateo et al., 2015; Hack et al., 2004) that is expressed in almost all cells cultured as neurospheres in the presence of bFGF and EGF (Dromard et al., 2007). It is therefore unsurprising that withdrawal of growth factors led to decreased Olig2 expression, and remained high in all growth factor-treated groups.

These results show the potential of dextrin-growth factor conjugates for further development as culture media supplements for *in vitro* stem cell expansion. Additionally, the results highlight the potential of biopolymer conjugation as an efficient and effective tool for localized, sustained delivery of growth factors to increase the survival, integration and differentiation of transplanted stem cells *in vivo*. Ongoing studies are evaluating dextrin-growth factor conjugates in *ex vivo* and *in vivo* models of SCI and investigating dextrin conjugation of other neural stem cell-specific growth factors.

## Author's contribution

**ELF**, **BS** and **DWT** conceived and designed the work. **ELF**, **SN**, **LCP**, and **JH** performed data collection and data analysis. **FY**, **BZ** and **QL** provided the primary neural stem cells (NSCs) and offered technical assistance with experimental design. All authors contributed to data interpretation. **ELF** and **SN** drafted the manuscript and all authors reviewed and approved the manuscript.

## Conflict of interests

The authors confirm that this paper content has no conflict of interests.

## Appendix A Supplementary data

S1: Proliferation of mNSC over 7 days. Growth curves (using the MTT assay) of cells grown as a monolayer, when cells were grown in growth factor-free medium (control), or in the presence of free- or dextrin-conjugated EGF and bFGF ±  amylase (100 IU/L). Data represent mean ± SEM, n = 18. S2: Proliferation of mNSC over 7 days. Growth curves (using the MTT assay) of cells grown as neurospheres, when cells were grown in growth factor-free medium (control), or in the presence of free- or dextrin-conjugated EGF and bFGF ±  amylase (100 IU/L). Data represent mean ± SEM, n = 18. Image 1
