# Supplementary material for: Controlled release of dextrin-conjugated growth factors to support growth and differentiation of neural stem cells
Source: Stem Cell Res. 2018 Dec;33:69–78. doi: 10.1016/j.scr.2018.10.008 (PMC6288241; doi:10.1016/j.scr.2018.10.008)
Supplement: Supplementary file 1 — S1: Proliferation of mNSC over 7 days. Growth curves (using the MTT assay) of cells grown as a monolayer, when cells were grown in growth factor-free medium (control), or in the presence of free- or dextrin-conjugated EGF and bFGF ± amylase (100 IU/L). Data represent mean ± SEM, n = 18. S2: Proliferation of mNSC over 7 days. Growth curves (using the MTT assay) of cells grown as neurospheres, when cells were grown in growth factor-free medium (control), or in the presence of free- or dextrin-conjugated EGF and bFGF ± amylase (100 IU/L). Data represent mean ± SEM, n = 18. [file mmc1.pptx]

## Slide 1
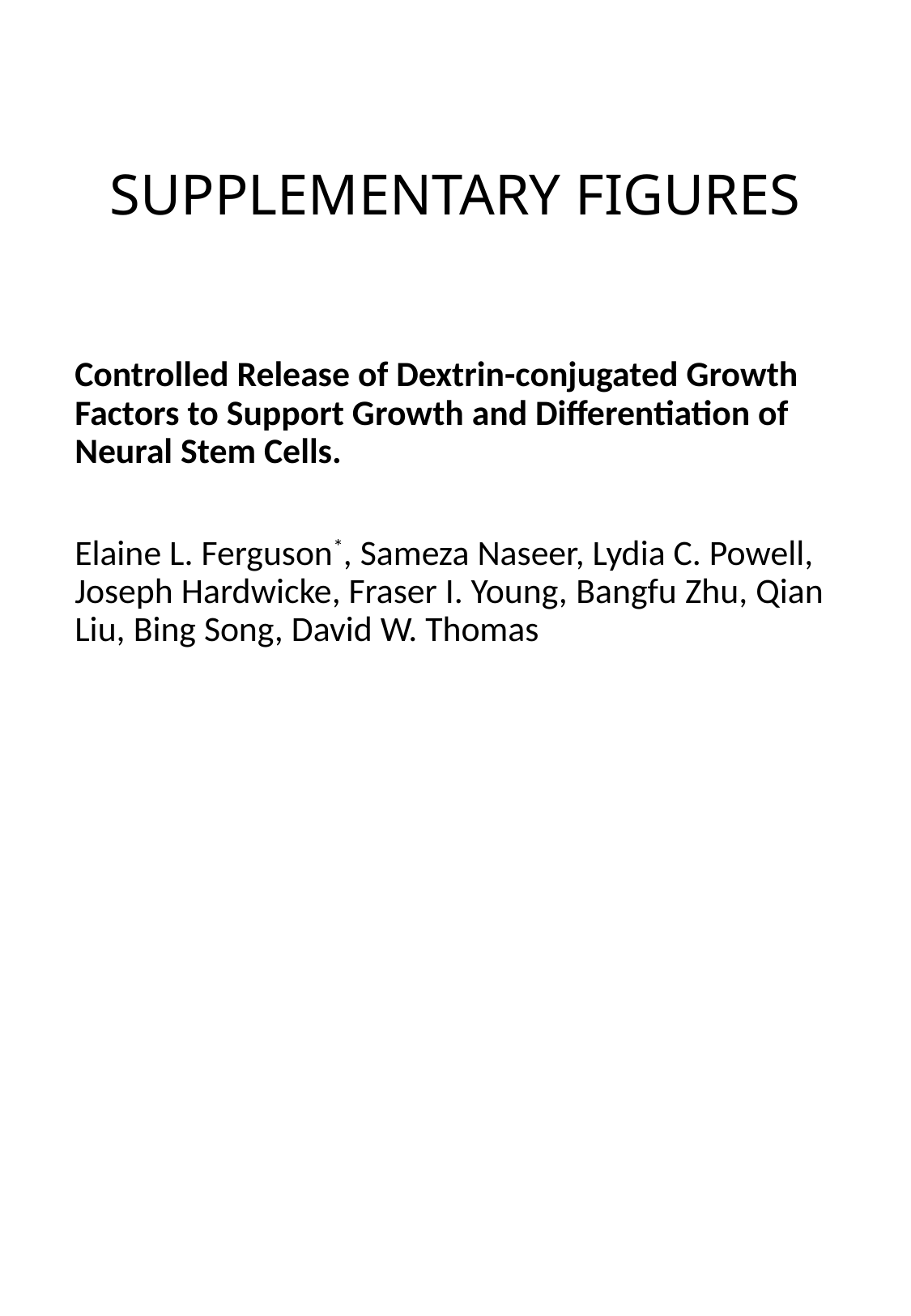

# SUPPLEMENTARY FIGURES
Controlled Release of Dextrin-conjugated Growth Factors to Support Growth and Differentiation of Neural Stem Cells.
Elaine L. Ferguson*, Sameza Naseer, Lydia C. Powell, Joseph Hardwicke, Fraser I. Young, Bangfu Zhu, Qian Liu, Bing Song, David W. Thomas

## Slide 2
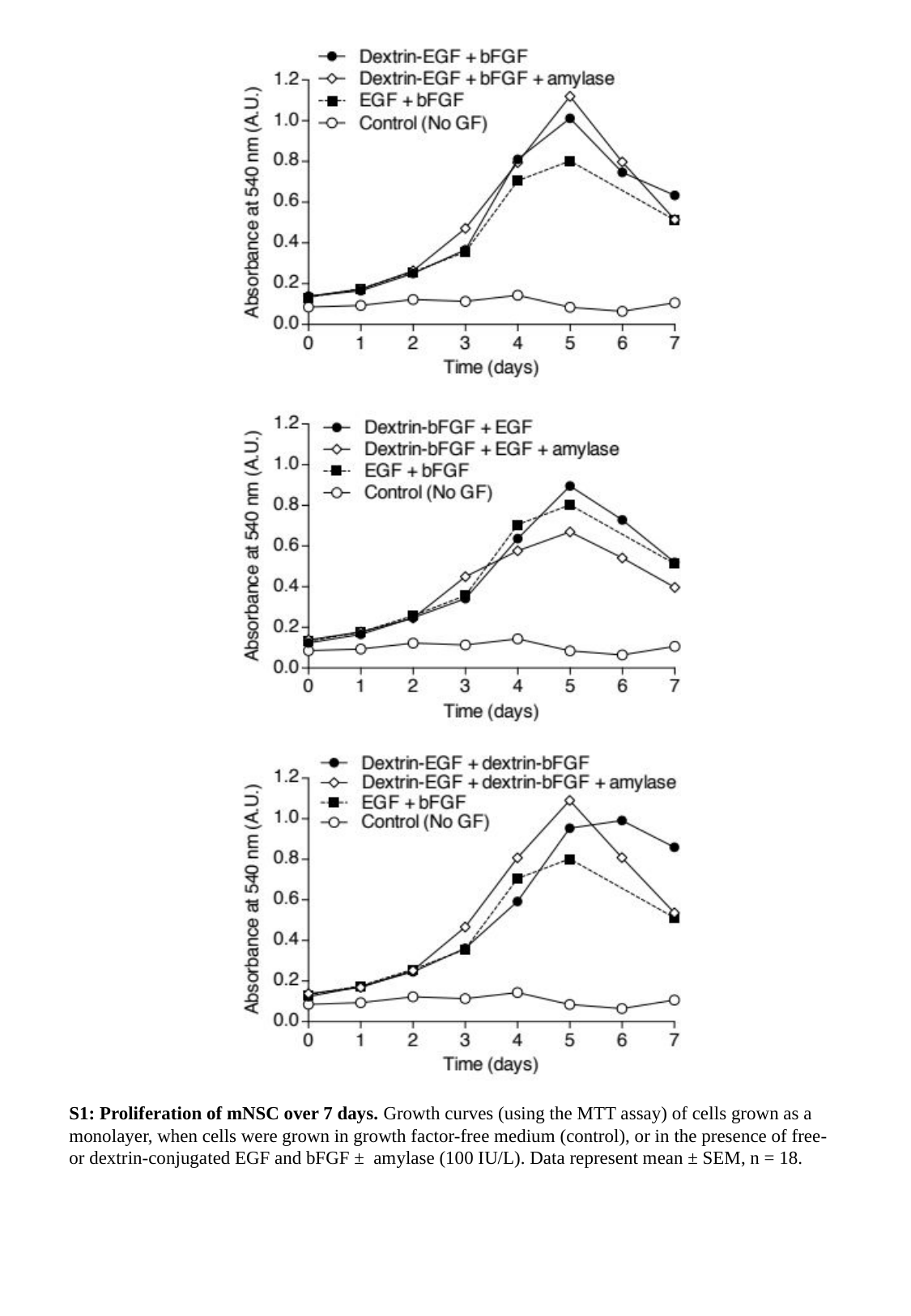

S1: Proliferation of mNSC over 7 days. Growth curves (using the MTT assay) of cells grown as a monolayer, when cells were grown in growth factor-free medium (control), or in the presence of free- or dextrin-conjugated EGF and bFGF ± amylase (100 IU/L). Data represent mean ± SEM, n = 18.

## Slide 3
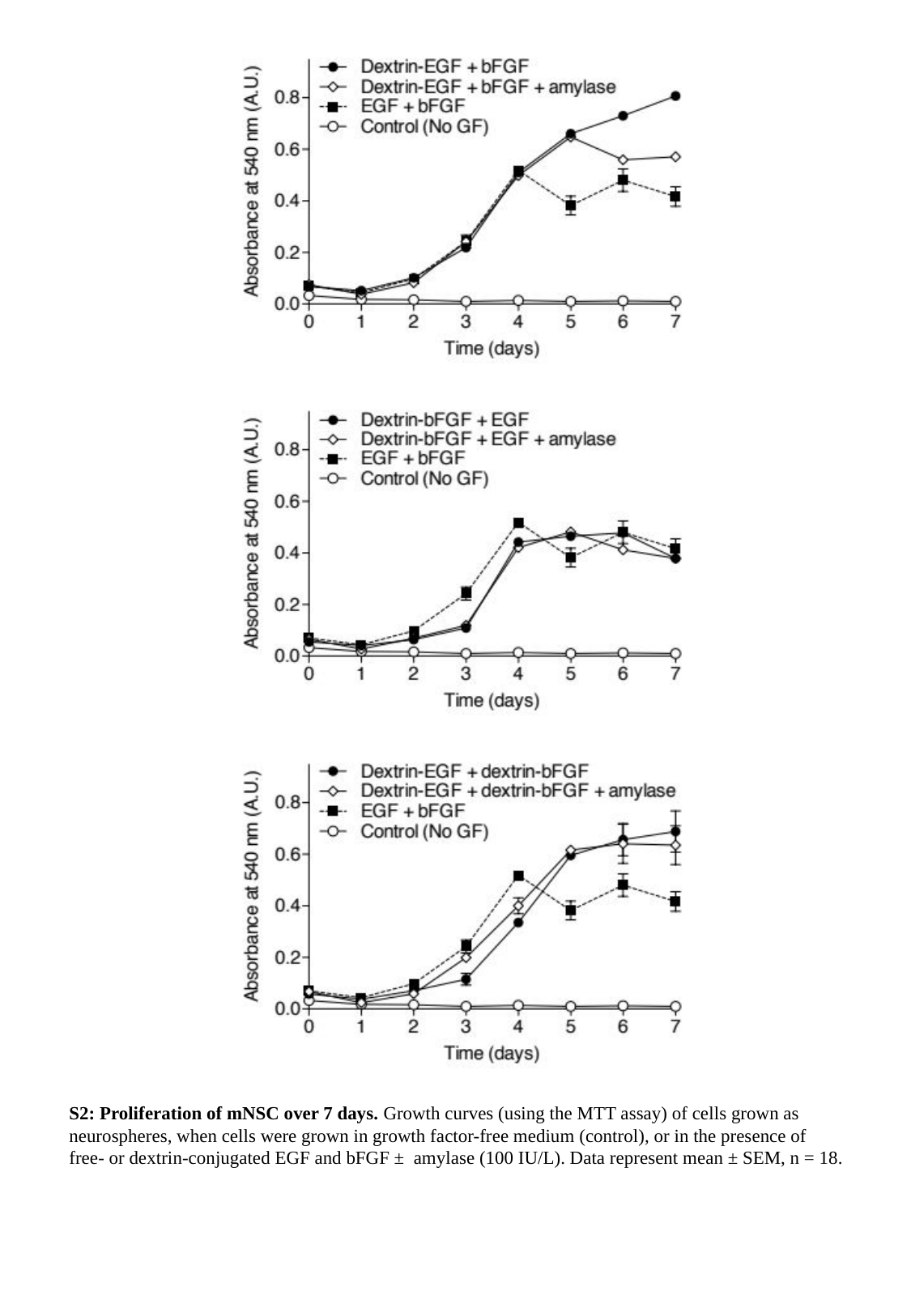

S2: Proliferation of mNSC over 7 days. Growth curves (using the MTT assay) of cells grown as neurospheres, when cells were grown in growth factor-free medium (control), or in the presence of free- or dextrin-conjugated EGF and bFGF ± amylase (100 IU/L). Data represent mean ± SEM, n = 18.
